# Supplementary material for: Consumption of Antibiotics and Epidemiology of Clostridioides difficile in the European Union in 2016—Opportunity for Practical Application of Aggregate ECDC Data
Source: Antibiotics (Basel). 2020 Mar 19;9(3):127. doi: 10.3390/antibiotics9030127 (PMC7148496; doi:10.3390/antibiotics9030127)
Supplement: Supplementary file 1 [file antibiotics-09-00127-s001.pdf]

Supplementary material, Table S1.

Healthcare-associated (HA) CDI, Community-associated (CA) CDI, antibiotic consumption in hospital/ community sector and quality indicators for antibiotic consumption in the community (primary care sector), 2016, the included European countries [6, 27,28].

Country	Healthcare-associated CDI	Community-associated CDI or unknown origin	Total	Consumption of antibacterials (ATC group J01) for systemic use in the hospital/community sector (2017)		Consumption of of quinolones (ATC group J01M) for systemic use in the hospital/community sector (2017)		Consumption of of other beta-lactam antibacterials (ATC group J01D) for systemic use in the hospital/community sector (2017)		Consumption of of macrolides, lincosamides and streptogramins (ATC group J01F) for systemic use in the hospital/community sector (2017)		Quality indicators for antibiotic consumption in the community (primary care sector) (2017)
	Mean hospital incidence density			Hospital	Community	Hospital	Community	Hospital	Community	Hospital	Community	Broad/narrow**
				DDD* per 1000 inhabitants and per day		DDD* per 1000 inhabitants and per day		DDD* per 1000 inhabitants and per day		DDD* per 1000 inhabitants and per day		
Austria	1.64	1.17	2.82	-	11.9	-	1.23	-	1.51	-	2.81	6.9
Belgium	1.93	0.84	2.78	1.64	21.1	0.183	2.17	0.363	1.17	0.109	3.41	123.92
Croatia	2.60	0.75	3.35	1.74	16.8	0.229	1.50	0.546	2.47	0.158	2.75	10.10
Estonia	12.93	1.88	14.81	1.65	9.9	0.201	0.79	0.549	1.18	0.161	2.26	14.69
Finland	3.61	0.70	4.31	2.11	13.6	0.217	0.67	0.838	1.94	0.124	0.75	0.5
France	2.52	1.60	4.12	2.13	29.2	0.191	1.37	0.330	1.60	0.118	3.04	47.17
Greece	3.10	1.03	4.12	2.29	37.4	0.257	2.62	0.575	7.73	0.133	6.98	123.00
Hungary	3.18	0.47	3.65	1.24	15.6	0.216	2.40	0.361	2.10	0.121	2.80	75.44
Ireland	3.02	1.01	4.02	1.60	19.3	0.098	0.81	0.186	1.08	0.237	4.19	4.65
Italy	2.27	0.50	2.76	1.89	19.0	0.388	2.68	0.394	1.94	0.184	3.75	208.48
Latvia	3.40	0.00	3.40	1.89	12.1	0.237	1.03	0.640	0.66	0.143	1.97	14.79
Lithuania	7.88	0.78	8.66	2.12	13.6	0.239	0.87	0.776	1.29	0.077	2.07	11.08
Malta	1.71	0.80	2.51	3.11	23.4	0.339	2.18	0.328	3.17	0.325	4.46	75.86
Netherlands	2.17	0.58	2.75	0.94	10.1	0.097	0.73	0.221	0.03	0.057	1.38	10.37
Poland	6.18	1.40	7.58	1.79	27.0	0.208	1.49	0.649	3.99	0.089	4.46	22.71
Slovenia	2.60	0.79	3.40	1.44	10.7	0.204	1.11	0.313	0.37	0.127	1.68	3.05
Spain	3.01	1.25	4.26	1.83	25.1	0.335	2.83	0.460	2.27	0.131	3.08	53.52
UK-Scotland	1.99	0.56	2.54	2.62	19.1	0.117	0.45	0.180	0.24	0.304	2.90	1.94
EU/EEA	2.38	0.81	3.19	2.03	21.8	0.230	1.60	0.390	2.00	0.16	2.90	

*DDD- defined daily dose

** Broad/narrow- ratio of the consumption of broad-spectrum antibiotic: combination of penicillins, including beta-lactamase inhibitor, second and third-generation cephalosporins, lincosamides and streptogramins (J01(CR+DC+DD+(F-FA01))) to the consumption of narrow-spectrum antibiotic: beta-lactamase-sensitive penicillins, first-generation cephalosporins and macrolides (J01(CE+DB+FA01)).
